# Population genomics of an endemic Mediterranean fish: differentiation by fine scale dispersal and adaptation

**DOI:** 10.1038/srep43417

**Published:** 2017-03-06

**Authors:** Carlos Carreras, Víctor Ordóñez, Lorenzo Zane, Claudia Kruschel, Ina Nasto, Enrique Macpherson, Marta Pascual

**Affiliations:** 1 Departament de Genètica, Microbiologia i Estadística and IRBio, Universitat de Barcelona, Av.Diagonal 643, 08028 Barcelona, Spain; 2 Department of Biology, University of Padova, via G. Colombo 3, 35131 Padova, Italy; 3 Consorzio Nazionale Interuniversitario per le Scienze del Mare, Piazzale Flaminio 9, 00196 Roma, Italy; 4 University of Zadar, Ul. Mihovila Pavlinovica, 23000 Zadar, Croatia; 5 Department of Biology, Faculty of Technical Sciences, Vlora University, 9401 Vlora, Albania; 6 Centre d’Estudis Avançats de Blanes (CEAB-CSIC), Car. Acc. Cala St. Francesc 14, 17300 Blanes Girona, Spain

## Abstract

The assessment of the genetic structuring of biodiversity is crucial for management and conservation. For species with large effective population sizes a low number of markers may fail to identify population structure. A solution of this shortcoming can be high-throughput sequencing that allows genotyping thousands of markers on a genome-wide approach while facilitating the detection of genetic structuring shaped by selection. We used Genotyping-by-Sequencing (GBS) on 176 individuals of the endemic East Atlantic peacock wrasse (*Symphodus tinca*), from 6 locations in the Adriatic and Ionian seas. We obtained a total of 4,155 polymorphic SNPs and we observed two strong barriers to gene flow. The first one differentiated Tremiti Islands, in the northwest, from all the other locations while the second one separated east and south-west localities. Outlier SNPs potentially under positive selection and neutral SNPs both showed similar patterns of structuring, although finer scale differentiation was unveiled with outlier loci. Our results reflect the complexity of population genetic structure and demonstrate that both habitat fragmentation and positive selection are on play. This complexity should be considered in biodiversity assessments of different taxa, including non-model yet ecologically relevant organisms.

The assessment of marine biodiversity, including genetic structuring, is one of the major goals of population management and conservation biology^1^. This assessment should ideally be achieved by the combination of two alternative approaches based on the analysis of neutral and adaptive loci^2^. The detection of barriers to dispersion is crucial in order to identify isolated units and to assess the degree of connectivity among populations. This detection is especially challenging for marine organisms, for which barriers to dispersal are less evident than those in the terrestrial environment and connectivity usually is due to larval stages^3^. Neutral genetic markers, such as microsatellites, have been extensively used for this purpose in the past decades^4^, but have lacked power to detect differentiation on several occasions due to recent divergence of populations, large population sizes, the limited number of markers used or homoplasy (e.g. refs 5,6). Another key process in evolutionary genetics is adaptation by natural selection that also drives population differentiation^2^. Local environmental conditions would also favour genetic differentiation among populations, especially considering that generally large-sized populations are more likely affected by selection than by genetic drift^7^. Furthermore, analysing the role of adaptation and the genes involved in the species’ response is necessary to ascertain the vulnerability of key species and populations under environmental change scenarios^8^. Studies on adaptation on natural populations have generally focused on specific and well known regions of the genome, like the MHC associated to the immune system^9^,^10^, heat-shock genes known to play a role in the stress response system^11^,^12^ or the genotypic component of phenotypic variation disentangled through common-garden experiments^13^,^14^. However, the combination of both neutral and selective markers to assess the distribution of genetic variability on non-model organisms is yet far to be common, especially in the marine realm, despite both types of markers provide complementary relevant information^2^,^15^.

Perhaps one of the flagships of the genomics era, favoured by high-throughput sequencing technologies, is the possibility to easily obtain Single Nucleotide Polymorphisms (SNP) markers in ecological-model species without reference genomes^16^,^17^,^18^. Although not exempt from problems (such as ascertainment bias), SNPs feature important advantages with respect to more ‘traditional’ markers such as microsatellites, including reproducibility between laboratories, high density of markers, and potential for annotation. One of the most important additional applications of SNPs developed by massive sequencing is the potential to study non-neutral signatures^19^, revealing adaptation processes when scanned along relevant environmental gradients^20^ or through the detection of outliers^21^. However, even in organisms with a reference genome, a method of genome reduction is necessary for species with medium to large genomes to ensure sequence depth for SNP identification. This identification is currently achieved using Restriction site Associated DNA sequencing (RAD-seq) protocols, among which the Genotyping-by-Sequencing (GBS) approach^22^ provides a cost effective methodology for high density SNP discovery and genotyping. Genome reduction techniques facilitate genotyping thousands of genetic markers simultaneously, even in non-model species, resulting in a recent expansion of population genomic studies^23^,^24^,^25^,^26^,^27^.

The CoCoNet European project (FP7 Actions) aims to establish Marine Protected Areas (MPAs) based on genetic data of key marine species and considered the Adriatic Sea as a Pilot Area^28^. The East Atlantic peacock wrasse (*Symphodus tinca*, Linnaeus, 1758) has several biological features that make this non-model organism a good candidate to study genetic structuring caused by both isolation and local adaptation. This demersal fish is considered a key species due to its abundance and generalist diet (sea-urchins, ophiuroids, bivalves, shrimps and crabs), being an important prey of large predators^29^, as well as constituting a common species in artisanal and spear fishing activities^30^. Furthermore it has a very short Pelagic Larval Duration (PLD), lasting only 9–13 days^31^, its larvae are never found more than a few hundred metres from the shore^32^. Adults exhibit territorial behaviour^33^ like most nest-building fishes^34^, and thus are considered to disperse only during the larval stages. The species lives mainly in shallow rocky shores with a high abundance of arborescent algae to build the nests^33^, which is common in the sampled localities of the Adriatic Sea Pilot Study^35^. Considering all these biological characteristics it is expected that the East Atlantic peacock wrasse would have a very limited dispersion range generating strong genetic population differentiation. The genetic structuring and the degree of connectivity between populations of this species had been studied along the western Mediterranean using eight microsatellites and, despite low dispersion predictions, only major discontinuities generated genetic differentiation^3^. This unexpected result could be attributed to the mating and settlement behaviour of the species^33^ or to the reduced number of analysed markers. High-throughput sequencing of genomic subsets targeted through restriction enzymes open the possibility of working on a genomic scale with non-model yet ecologically relevant species, like *S.tinca*^22^.

The aim of this study was to assess connectivity among present and future Marine Protected Areas (MPAs) within the southern Adriatic and northern Ionian Seas to identify the effect of different types of markers in determining genetic differentiation. More specifically, using Genotyping by sequencing we 1) genetically characterized 176 individuals of the East Atlantic peacock wrasse (*Symphodus tinca*) from 6 locations all of them within existing or planned MPAs and 2) identified putatively neutral loci and positively selected loci to determine changes in population genetic structure based on these two sets of markers. Finally, we discuss the implications of our results and the potential of this approach for the study of non-model organisms thus showing how these new genomic approaches can be applied to marine molecular evolutionary studies and the design of networks of MPAs.

## Results

**Figure 1:**
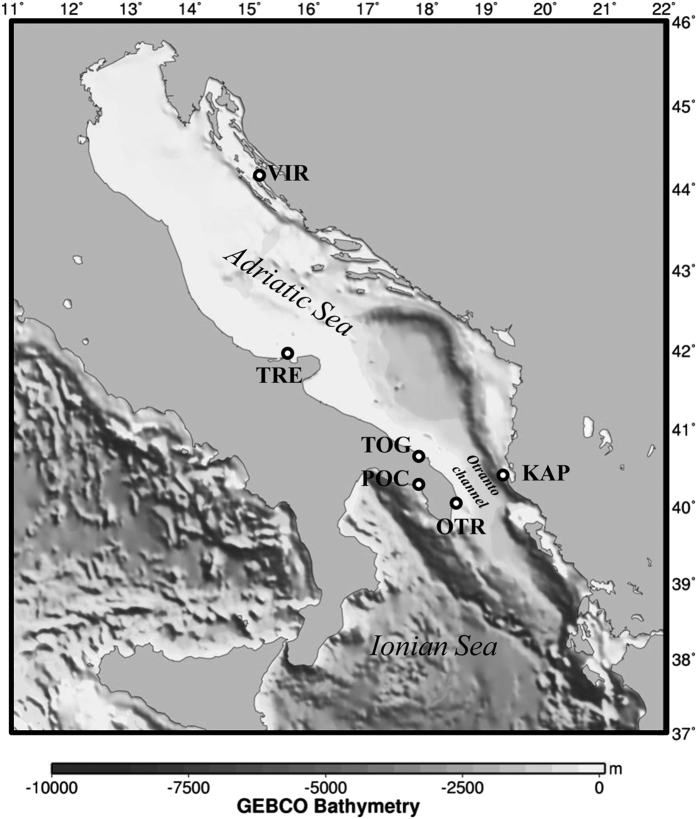
Sampling locations of *Symphodus tinca* within the Adriatic and Ionian Seas. See Table 1 for detailed description of each location. Map created using the free software MAPTOOL (SEATURTLE.ORG Maptool. 2002. SEATURTLE.ORG, Inc. http://www.seaturtle.org/maptool/ 30 July 2015), that uses GMT (The Generic Mapping Tool)^81^. KAP: Karaburun Peninsula, Albania; VIR: Island of Vir, Croatia; TRE: Tremiti Islands, Italy; TOG: Torre Guaceto, Italy; OTR: Otranto, Italy and POC: Porto Cesareo, Italy.

The 176 samples of *Symphodus tinca* analysed by GBS from the Adriatic and Ionian seas (Fig. 1, Table 1) were sampled in Karaburun Peninsula, Albania (KAP, n = 28), Island of Vir, Croatia (VIR, n = 35) and the Italian sites of Tremiti Islands (TRE, n = 22), Torre Guaceto (TOG, n = 32), Otranto (OTR, n = 29) and Porto Cesareo (POC, n = 30).

### General SNP calling and filtering

We obtained a total of 440.7 million high-quality reads, with a mean of 2.5 million reads per individual that resulted in a total of 231,884 paired tags for all samples. A mean of 383,502 reads per individual were correctly mapped against the previously defined paired tags used as reference (Table 1). A total of 51,221 putative SNPs were identified among our samples and 4,155 polymorphic SNPs retained over all samples after applying all filters (see methods section, Supplementary Data S1). The mean read depth per individual was 39.8 reads per locus (SD = 12.7 across individuals, SD = 23.4 across loci). The number of polymorphic SNPs was positively correlated to sample size (r = 0.89, P = 0.016). The number of alleles and the observed and expected heterozygosities were positively and negatively correlated to sample size respectively. These correlations became non-significant when we corrected these parameters by sample size and the total number of SNPs respectively (Table 1).

### Population genomics

Most pairwise comparisons among sampling locations were significantly different using both F_ST-WC_ and F_ST-RH_ estimators (Table 2), with the exceptions of Porto Cesareo (POC) versus Torre Guaceto (TOG) and Vir (VIR) versus Karaburun (KAP). The two estimators were significantly correlated as assessed with a Mantel test (r = 0.99, p = 0.020). However, two additional pairwise comparisons were significant with F_ST-RH_ but not with F_ST-WC_ (Otranto-OTR versus POC and OTR versus TOG), probably due to F_ST-RH_ performing better at low or moderate levels of differentiation^36^. With both estimators we could define three different units (Table 2). Tremiti (TRE) was the most differentiated location and the locations from the eastern shore of the Adriatic (VIR and KAP) were in all cases different from the south-western locations (TOG, POC and OTR).

**Figure 2:**
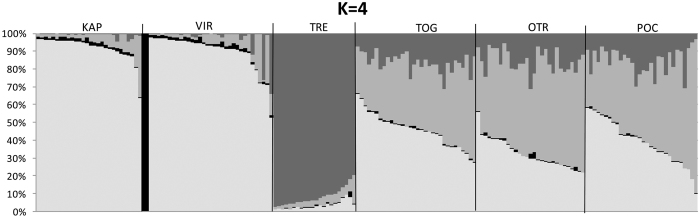
Posterior probabilities of individual assignment to the most probable number of clusters (K = 4). Each bar represents one individual. KAP: Karaburun Peninsula, Albania; VIR: Island of Vir, Croatia; TRE: Tremiti Islands, Italy; TOG: Torre Guaceto, Italy; OTR: Otranto, Italy and POC: Porto Cesareo, Italy.

**Figure 3:**
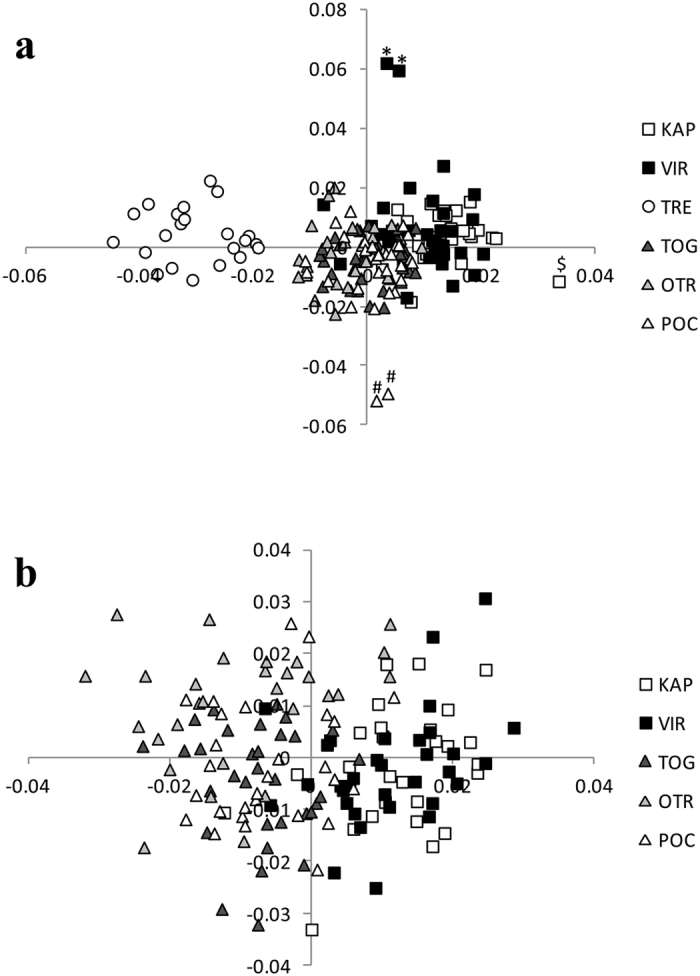
Multi Dimensional Scaling (MDS) plots of the 176 individuals of *Symphodus tinca* using all SNPs. (**a**) including all individuals, (**b**) excluding all individuals from Tremiti, the outliers from Vir (*), Porto Cesaeo (#) and Karaburun ($). KAP: Karaburun Peninsula, Albania; VIR: Island of VIR, Croatia; TRE: Tremiti Islands, Italy; TOG: Torre Guaceto, Italy; OTR: Otranto, Italy and POC: Porto Cesareo, Italy.

The most likely number of populations identified by STRUCTURE was four (K = 4) as identified by ΔK (Supplementary Fig. S1). The probability of assignment of each individual to each of these groups (Fig. 2) revealed an overall differentiation of Tremiti from the eastern and the south-western locations but also differentiated two individuals from Vir. The MDS analysis including all individuals (Fig. 3A) clearly separated with the first axis all Tremiti individuals. Furthermore, five additional individuals were also separated from the remaining bulk of samples, one individual from Karaburun, the two from the Island of Vir already detected by STRUCTURE and two from Porto Cesareo. These five individuals had similar mean number of reads and SNP missingness than the other samples, so apparently its divergence was not a technical artefact. In order to clarify the structuring of the remaining samples we repeated the MDS analysis without the individuals from Tremiti and the five divergent samples. With this approach we found a much clearer separation of individuals sampled in eastern and south-western populations along the first axis although intermixing was observed for some populations (Fig. 3B). The assignment analyses showed that only around half of the individuals were self-assigned to the sampling locations with the exception of Tremiti, where almost all individuals were correctly self-assigned (Supplementary Table S2). However, we repeated the analysis under consideration of the three genetically different groups identified with F_ST_ values (Table 2), now constituting the putative populations (Tremiti, eastern locations and south-western locations), with the consequence that almost all individuals of all locations were correctly assigned to the corresponding population (Supplementary Table S2). All five divergent samples were assigned to the corresponding sampling locations and groups.

### Detection of outlier SNPs

From the 4,155 polymorphic SNPs found in our samples, 78 significant outlier SNPs were detected by ARLEQUIN after FDR correction (Supplementary Fig. S2), all of them potentially under positive selection (F_ST_ > 0.05). Without applying FDR correction 3,934 SNPs were assumed to be neutral as they were not significantly under selection, although neutrality cannot be directly proven. Finally, the remaining 143 SNPs were not classified in any of the former categories as yielded significant p-values but only before FDR correction. No outlier SNP was identified as to be under balancing selection but preliminary results, without filtering by a mean depth per genotype higher than 100X, identified 59 SNPs under balancing selection. Thus read depth has to be considered before assigning SNPs to any given category since the existence of paralog genes in the genome might erroneously identify SNPs as putatively being under balancing selection. BAYESCAN identified 19 statistically significant outlier SNPs, all of them potentially under positive selection, and all of them already detected by ARLEQUIN.

**Figure 4:**
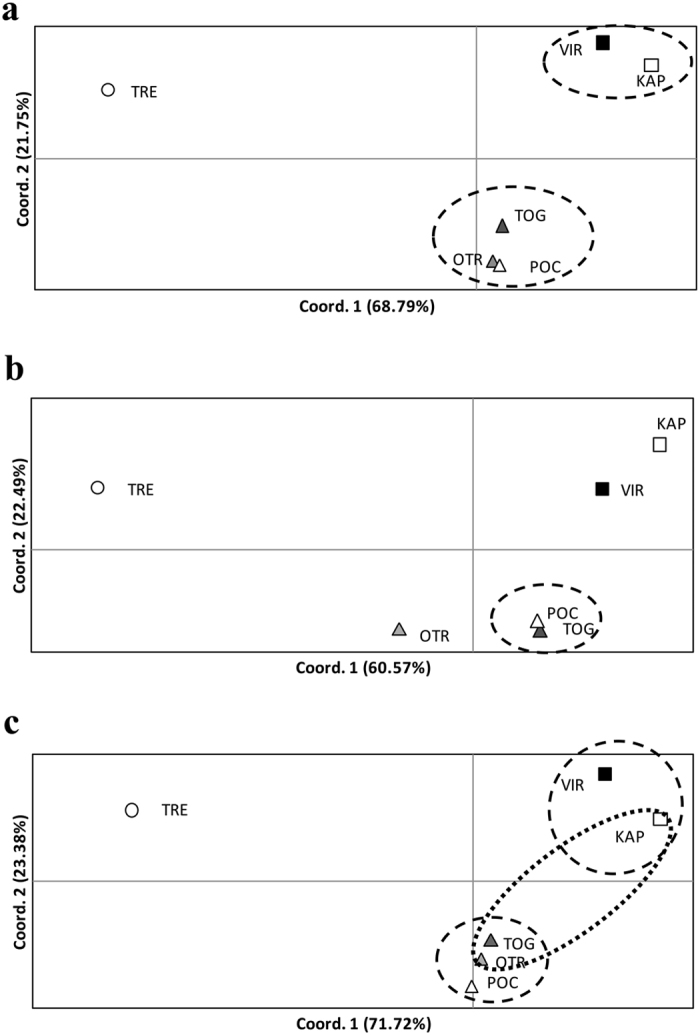
Principal Coordinate Analysis (PCoA) of S*ymphodus tinca* locations of the Adriatic Sea using F_ST_ pairwise genetic distances. (**a**) using the 4,155 polymorphic SNPs, (**b**) using the 19 outlier SNPs under local adaptation and (**c**) using the 3,934 neutral SNPs. The percentage of variability explained by each coordinate is shown in brackets. Dashed lines encircle the non-significantly differentiated locations after FDR correction as in Table 2 and Table S1. Dotted line encircle locations non-significantly differentiated but with p-values close to significance as in and Table S1. KAP: Karaburun Peninsula, Albania; VIR: Island of Vir, Croatia; TRE: Tremiti Islands, Italy; TOG: Torre Guaceto, Italy; OTR: Otranto, Italy and POC: Porto Cesareo, Italy.

**Figure 5:**
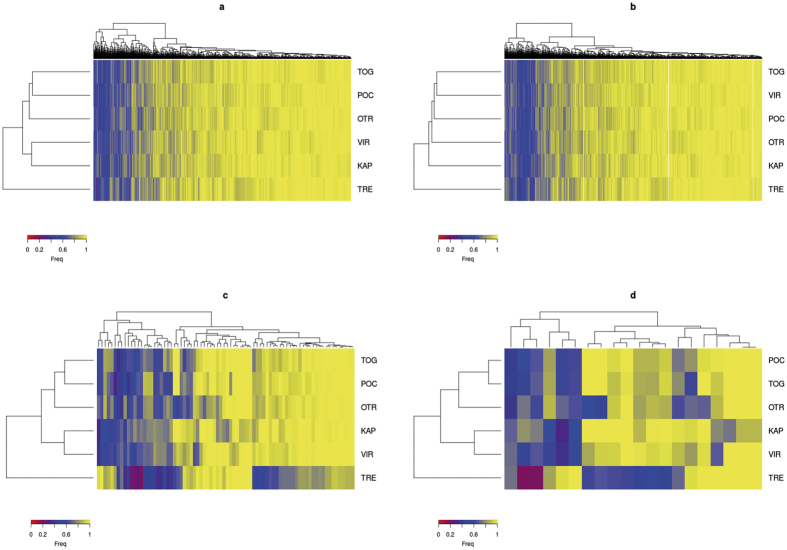
Heatmap of the major allele frequency for each SNP in the six populations of *S. tinca.* (**a**) considering the 4,155 polymorphic SNPs, (**b**) considering the 3,934 neutral SNPs, (**c**) considering the 78 outlier SNPs obtained from coalescent simulations and (**d**) considering the 19 outlier SNPs obtained from bayesian simulations. Dendrograms group population on the left axis and SNPs on the top axes according to frequency similarity. KAP: Karaburun Peninsula, Albania; VIR: Island of Vir, Croatia; TRE: Tremiti Islands, Italy; TOG: Torre Guaceto, Italy; OTR: Otranto, Italy and POC: Porto Cesareo, Italy.

Pairwise F_ST-WC_ comparisons showed very different values when only outlier or neutral SNPs were used (Supplementary Table S1). As expected, the 19 outlier SNPs, detected by ARLEQUIN and BAYESCAN to be potentially under positive selection, showed higher F_ST-WC_ values than when using all the SNPs. Furthermore, all pairwise comparisons with only outlier loci were significant but one (Porto Cesareo versus Torre Guaceto). Neutral SNPs also showed significant structuring, but with lower F_ST-WC_ values than the whole set of SNPs, and one more non-significant value involving the locations across the Otranto channel: Karaburun Peninsula and Otranto (Supplementary Table S1, Fig. 1). Despite these differences, both sets of pairwise F_ST_ values were significantly correlated as assessed with a Mantel test (r = 0.927, P = 0.01). Regardless of the set of SNPs used similar population distribution was obtained with a PCoA analysis, which roughly reflected the geographical position of the sampled locations (Fig. 4). The first axis, explaining around 70% of the differentiation in all set of SNPs, clearly differentiated Tremiti from the other locations while the second axis, explaining roughly 20%, separated the south-western from the eastern locations (Fig. 4). Sampling locations were similarly grouped by the heatmaps based on their allele frequency distribution, although groupings were more clearly differentiated when using any of the two sets of outlier markers (Fig. 5). A MDS performed for all individuals using only neutral SNPs revealed that four of the five divergent individuals detected using the whole set of SNPs (those two from Vir and those two from Porto Cesareo) were also divergent for neutral SNPs (Supplementary Figure S3).

The 78 sequences with SNPs identified as outlier with either ARLEQUIN or BAYESCAN were blasted against the genome of the Nile tilapia (*Oreochromis niloticus*), and 12 of them yielded significant matches (Supplementary Table S3). Four were located within a known gene (minimum distance between the gene and the SNP equal to zero): three of them completely within an intron and one sequence (SNP S1_2262059, Supplementary Table S3) overlapped exonic and intronic regions of the neuronal pentraxin 2b gene (nptx2b). The outlier SNP of this sequence was a T/A located seven nucleotides upstream of the exon before a polypyrimidine tract. An A in this position, as described in the literature, seems to be involved in the branch point of the lariat formation during intron removal^37^. Thus the T change could compromise the correct splicing of intron two of nptx2b. The function of this gene was related to the regulation of circadian rhythm^38^ suggesting that adaptation of this SNP could be related to environmental factors. The frequency of the allele that allowed the lariat formation (A) apparently increased with latitude and was higher in the western part of the Adriatic, although the correlation had a low number of observations (Supplementary Fig. S4).

## Discussion

The recent incoming of new genomic tools based on next-generation sequencing is revolutionising molecular ecology and evolutionary studies, especially applied to non-model organisms. They not only outperform traditional markers, but also open new research opportunities including the role of adaptation in population differentiation^24^,^27^. In this study we used GBS to study the genetic structure of an ecologically relevant Mediterranean endemic fish, for which traditional markers had revealed a genetic structuring not consistent with the known biology of the species (e.g. low dispersal capabilities), thus showing how these new genomic approaches could change our vision of marine molecular ecology and evolution.

Using 8 microsatellites, Galarza *et al*.^3^ found significant genetic structuring in *Symphodus tinca* populations only along the Almeria-Oran Front, an oceanographic discontinuity in the west Mediterranean that has been reported to be a barrier to gene flow for a high number of species^39^. However, no trace of genetic differentiation was found for *S. tinca* along other known fronts, like the Balearic Front, which isolates other fish species with much longer PLD and with an offshore larval distribution^3^. Even though we have not sampled the same populations, and thus a direct comparison is not possible, we have found significant genetic structure among populations separated by smaller distances and depths than in the previous study, suggesting that GBS provides more resolution when assessing genetic differentiation given that thousands of loci are being assessed. Likewise, recent studies on marine organisms have found deeper levels of genetic differentiation using genome-wide approaches rather than using traditional genetic markers e.g. ref. 25 and it has been proposed that thousands of markers at genome level are often required to distinguish among alternative scenarios, for instance when reconstructing invasion histories^40^. In our study we have analysed up to 14 Mb of the genome considering the ~250 K paired tags detected among all samples. This length would roughly correspond to a 2% of the *S. tinca* genome^41^. Given that 94.5% of our SNPs would be considered neutral, our study also indicates that analysing thousands of curated SNPs is necessary to identify loci potentially under local adaptation in addition to identify connectivity patterns assessed from the neutral markers. Furthermore our results call for caution when SNPs are identified to be under balancing selection since a higher number of reads than expected would suggest the presence of paralogs.

Previous studies within the Adriatic Sea did not find relevant barriers to dispersal in many marine organisms, including fishes^42^,^43^,^44^. However, some studies demonstrated a weak north-south differentiation^45^,^46^,^47^. These findings agree with the hydrodynamic provinces suggested in the Mediterranean from Lagrangian simulations and network reconstruction identifying different regions in the Adriatic Sea according to different PLDs^48^. Similarly, Lagrangian simulations assuming larval movement of *Carcinus aestuarii* support the oceanographic subdivision of the Adriatic Sea into three sub-basins matching currents from north to south^49^. However, only weak genetic differentiation was observed in *C. aestuarii* mostly differentiating northern and southern locations along the western coast. According to these Lagrangian simulations, our sample from Tremiti could be considered to be representative of a central group of *S. tinca* for its geographical location, and thus it would explain its strong genetic differentiation from the south-western localities independent of the set of SNPs. A clear genetic break was also observed for *Aphanius fasciatus* using mtDNA in the eastern Adriatic coast, and at approximately the same latitude of Tremiti, that was attributed to a divergence process during the Pleistocene^50^. However, our populations along the eastern Adriatic coast were not strongly differentiated thus suggesting that the subdivisions might vary comparing the east to the west and the marker used. Most interestingly two individuals found in Vir and two in Porto Cesareo were genetically differentiated and unlikely to belong to the populations sampled in the present study. One possible explanation could be that these individuals represent recent migrants from north-eastern populations or from the central Ionian Sea, respectively. These areas, not included in the present study, have been suggested to be differentiated according to Lagrangian simulations^48^ and thus future sampling of additional locations in these northern and southern areas would help to clarify how many genetically differentiated groups are within the Adriatic and their connections. A recent study using biophysical modelling on *Symphodus ocellatus*, a species with similar larval characteristics as *S. tinca*, suggested a high larval retention and confined dispersal across a narrow geographic range, with occasional and weak connections across the two shores of the Adriatic^35^. This suggested that high larval retention is in accordance with our results since even using only neutral SNPs we detected north-south dispersal limitations and an east-west barrier for *S.tinca* within the Adriatic Sea. A recent mtDNA and microsatellite study on the black scorpionfish (*Scorpaena porcus*) in the Adriatic Sea sampling similar localities found some east-west differentiation although was not fully supported by all pairwise comparisons^28^. The differences with our study could be due to the different resolution of the molecular markers used or to real differences in connectivity mediated by a longer larval duration among others. Therefore, genome-wide SNP genotyping could provide much greater power than traditional markers to detect genetic differentiation and thus, to define barriers to the dispersion of studied organisms^51^,^52^.

One of the most exciting advantages of the genomic approach, compared to traditional markers, possibly relies on the chance to identify traces of selection that shape population genetic differentiation^2^. This advantage is especially promising considering that the role of selection has often been ignored in biodiversity assessments. The assessment of the structuring driven by selection is not unprecedented, as several studies have found significant genetic structure when analysing markers under selection, contrasting to a lack of structuring when using neutral markers^23^,^24^,^53^. This apparent contradiction has been linked to the generally large effective population sizes of the study organisms and an associated minimal effect of genetic drift. Under large-population scenarios, allele frequencies of different populations are expected to change mainly by differential selection pressures, and even under high levels of migrants, the resulting gene flow would not be sufficient to dilute the genetic differentiation of these markers generated by selection^24^. Our study detects differentiation using different sets of SNPs, outlier and neutral markers, although the signal greatly reduces for the latter. Thus, the resulting genetic structure reflects the effect of two different components, dispersion and selection, and both should be considered when defining units for management and conservation^2^.

A complex picture of the genetic structure of *Symphodus tinca* within the Adriatic Sea emerges as a result of the conjunction of these two components. The dispersal potential of this species, likely determined by larval characteristics^31^,^34^, limits its connectivity over long distances and across significant depths. The adaptation to local conditions may strengthen even more this differentiation and thus this study supports previous work hypothesizing that natural selection shapes marine populations at much smaller scales than expected (e.g. refs 23, 54 and 55). Some clues of the elements of selection defining this local adaptation could be found by comparing the sequences of the outlier SNPs to whole genomes. Unfortunately most of the outlier loci (85.2%) did not yield significant matches with the closest available sequenced genome represented by the Nile tilapia. Furthermore, the detection of outlier SNPs does not necessarily mean that these SNPs are located in the gene influenced by the selection. For instance most of our outlier SNPs were located on intergenic regions, probably reflecting linkage disequilibrium to a neighbouring candidate gene or regulatory region. Furthermore, our species lacks a reference genome, and thus the linkage between SNPs and neighbouring genes assumes synteny across species, which is something that remains to be tested. For all these reasons, our results should be interpreted with caution and only as general evidences of the spectrum of elements which could be affecting local adaptation. However, some evidence was obtained for SNP S1_2262059 located within a circadian gene, as it matched a regulatory position involved in intron splicing and its frequency varied with latitude. The information obtained through the comparison of the sequences containing the outlier SNPs with a related genome could be helpful when designing future studies, for instance Genome-Wide Association studies GWAs^56^, or when targeting specific genes.

The somewhat different results found when using different sets of markers open the debate of what constitutes the correct set of markers to use when inferring both genetic structuring and connectivity. While F_ST_ values obtained by different sets of markers correlated, showing that the relative genetic distances among localities were maintained, the significance of the comparisons obtained from these sets were slightly different. The neutral set of markers showed moderate levels of connectivity between the Adriatic populations across the Otranto channel while being highly differentiated with the outlier loci. This may either suggest that population structure is driven by selection, or that outlier SNPs are simply loci that show higher differentiation because they were picked on the upper end of an F_ST_ distribution caused by genetic drift. If we assume that outlier SNPs are under selection, we then should include them to infer genetic structuring to delineate conservation and management units^2^. Furthermore, outlier SNPs should ideally not be included to infer connectivity levels among populations, as even highly connected populations may show signals of genetic differentiation due to selection. Thus the inclusion of outlier SNPs may result in an underestimation of the levels of migration among populations^53^. In our case-study, either considering neutral or outlier SNPs potentially under positive selection the differentiation at both sides of the Adriatic Sea and in a north-south axis seem clear. The major differences between the different types of SNPs exist across the Otranto channel, at the entrance of the Adriatic. Thus, although the individuals may physically cross through this narrow strait from East to West and thereby mix the populations at both sides of the Adriatic, as suggested by neutral SNPs, the outlier loci results indicate that local conditions may be different enough to prevent the genetic homogenisation of these populations through selection. A similar situation has been suggested across the Atlantic-Mediterranean transition, were temporal fluctuations suggests a complex balance of dispersal and selection^57^. Consequently, the differences found in outlier loci should also be taken into account when defining management units for this species because the degree of genetic structuring is clearly deeper than suggested by only neutral markers. Particularly, managers should consider the populations at both sides of the Otranto channel as different units, regardless of the small geographic distance among them, because each area holds a particular set of locally adapted genes. These results are especially relevant when planning networks of Marine Protected Areas (MPAs) and provide insights about how these MPAs are connected considering that Karaburun Peninsula, Tremiti, Torre Guaceto and Porto Cesareo are extant MPAs and Otranto is planned to be a MPA. All south-western MPAs would form an MPA network that would be connected to eastern Adriatic MPAs, as shown by neutral SNPs, but genetically different due to positive selection, as shown by outlier SNPs (Fig. 4). Furthermore, these connections seem not to be symmetrical, as Torre Guaceto, Vir and Porto Cesareo export migrants, while Karaburun Peninsula and Otranto located at both sides of the Otranto channel mostly receive migrants. Considering this asymmetry, protection of source populations should be a priority for management and conservation plans considering their role as network builders. Finally Tremiti MPA would be independent to all of our other localities and could be considered to belong to a different cell of ecosystem functioning^58^.

Overall, our study clearly indicates that GBS is a good approach for population genomic studies of non-model organisms and emphasizes the novelties it brings, particularly to the study of marine organisms. Population genomics studies, as inferred through neutral and outlier SNPs, increase the ability to identify genomic areas under selection which then enhances our knowledge on how dispersal and local adaption shape biodiversity structuring.

## Methods

### Sampling

Samples of *Symphodus tinca* were obtained from 6 different locations within the Adriatic and Ionian seas (Fig. 1, Table 1). Tissue samples or fin clips were taken from adults captured by fishermen using spear fishing. Samples were taken from June 2013 to February 2014 and stored in 96% Ethanol. The collection of fish samples was conducted in strict accordance with Spanish and European regulations. The study was found exempt from ethics approval by the ethics commission of the University of Barcelona since, according to article 3.1 of the European Union directive (2010/63/UE) from the 22/9/2010, no approval is needed for fish sacrification with the purpose of tissue or organ analyses. Furthermore, the study species *Symphodus tinca* is not listed in CITES.

### Laboratory procedures

DNA was extracted from samples using the QIAamp^®^ DNA Mini Kit (QIAGEN) extraction kit following manufacturer’s instructions. DNA integrity was checked by gel electrophoresis, quantified by NanoDrop^®^ and 1.5–3 μg of DNA per sample was sent to the Cornell University Biotechnology Resource Centre (BRC) to perform GBS^22^. At the Cornell BRC Genomic Diversity Facilities, individual libraries were produced after digestion with EcoT22I and ligation of a barcode adaptor and a common adaptor with appropriated sticky ends. A total of 95 samples and a blank sample per plate were pooled and cleaned using the Qiagen PCR cleanup kit^®^ following manufacturer’s instructions. The two resulting 96plex libraries were then amplified by PCR using generic primers matching the adaptors and the following PCR conditions: 5 minutes at 72 °C, 30 seconds at 98 °C, 18 cycles of 10 seconds at 98 °C, 30 seconds at 65 °C and 30 seconds at 72 °C and a final extension of 5 minutes at 72 °C. The PCR was cleaned with the QIAquick PCR Purification kit^®^, diluted and single-end sequenced in an Illumina HiSeq 2500 platform at BRC, by using one lane per plate and the HiSeq v4 reagents kit.

### SNP calling

Raw sequences from Illumina were used for genotyping using the UNEAK pipeline^59^ as implemented in Tassel vs 3.0^60^. All data from different plates were analysed simultaneously using a common keyfile after removing the blank samples. All high-quality reads with a corresponding barcode were trimmed to 64 bp, excluding primers and barcodes, and all identical reads were merged as unique tags. Resulting tags were pairwise-aligned and all the possible pairs combined as networks. Only reciprocal tag pairs with a 1 bp mismatch were retained as potential SNPs after filtering with a certain tolerance error (set to 0.03). Reads from each individual were then mapped against the retained paired tags to extract the individual genotypes. VCF files were generated by applying a filter to a maximum number of 2 alleles per SNP. Additional filters were then applied using VCFtools vs 1.12^61^. We first filtered the individual genotypes by retaining only those with a minimum depth of 5X and a genotype quality (GQ) higher than 98. We also removed SNPs with a minimum allele frequency (MAF) lower than 0.01 and a missingness value higher than 30% (retaining SNPs present at >70% of the individuals). Finally, we removed SNPs with a mean depth per genotype higher than 100X to avoid possible paralogs since preliminary results without removing them yielded a high number of SNPs with large number of reads identified to be under balancing selection.

### Population genomics

VCF files were converted to PLINK vs 1.9^62^ using VCFtools. Additionally were also converted to ARLEQUIN vs 3.5^63^, GENETIX vs 4.05.2^64^, STRUCTURE vs 2.3.4^65^, BAYESCAN vs 2.1^66^ and GeneClass2 vs 2.0^67^ using the file converter PDGSpider vs 2.0.8.3^68^. ARLEQUIN was used to check for departure from Hardy-Weinberg Equilibrium and all loci deviating in at least the 60% of the localities were removed from further analyses^25^. ARLEQUIN was also used to calculate general diversity indices for each location and for computing F_ST-WC_ pairwise population values^69^. Allelic richness was calculated using the software ADZE vs 1.0^70^. Genetic differentiation was also assessed using the corrected^36^ values of F_ST−RH_^71^ with GENETIX. These two different F_ST_ measures were used as F_ST-WC_ is recommended for high values of differentiation and F_ST−RH_ for low or moderate values of differentiation^36^. A FDR correction for multiple comparisons was applied to calculate the appropriate threshold of differentiation^72^. Population structuring was also evaluated using the programme STRUCTURE, which implements a Bayesian clustering method to identify the most likely number of genetically differentiated populations (K). We used the strategy and parameters described in the literature^73^ and thus we carried out 10 runs per each value of K ranging from 1 to the number of localities plus two. We used the model of correlated allele frequencies and a burnin of 50,000 followed by 500,000 Markov Chains Monte Carlo. We estimated the ad hoc statistic ΔK in order to infer the most likely number of populations using STRUCTURE HARVESTER^74^, The 10 runs of STRUCTURE for the most probable K were averaged using CLUMPP vs 1.1.2^75^. A Multi-Dimensional Scaling (MDS) analysis was performed for all individuals using PLINK and the results were plotted using an Excel^®^ spreadsheet. We also tested if all individuals were correctly reassigned to their sampling locations by using GeneClass2^67^ that implements the Bayesian approach described in the literature^76^ and excludes the individual from their population during computation (leave-one-out procedure). Only the individuals with an assignation score higher that 95% were considered to be correctly assigned.

### Detection of outlier SNPs

We identified outlier SNPs using two different programs, ARLEQUIN and BAYESCAN. ARLEQUIN uses coalescent simulations to create a null distribution of F-statistics and then generates P-values for each locus based on its distributions and observed heterozygosities across all loci^21^. We considered each location as a unit to implement a hierarchical island model in order to reduce false positives introduced due to population structure. We performed a total of 20,000 simulations, 10 simulated groups and 100 demes per group. This method detects outlier SNPs with high F_ST_ values, considered to be potentially under positive selection, and outlier SNPs with F_ST_ values close to zero, considered to be candidates for balancing selection. To reduce the error due to multiple comparisons we applied a FDR correction^72^ to identify statistically significant outlier SNPs. However, corrections for multiple pairwise comparisons dramatically increase the probability of type II error (β: e.g. assume neutrality of a SNP when it is really not neutral), an effect that becomes worse as many P-values are discarded^77^. For this reason, we followed a conservative approach and we did not apply any correction to identify putatively neutral markers. Additionally, we identified outlier SNPs using BAYESCAN^66^. This software uses a Bayesian approach to estimate population specific F_ST_ coefficients in contrast to a locus-specific F_ST_ coefficient shared by all the populations. When the locus-specific component is needed to explain the observed pattern of diversity, the software assumes departure of neutrality either due to positive selection or to balancing selection. We run 100,000 simulations and specified a prior odd of 10,000 in order to minimize false positives^78^. We considered outlier SNPs those with a q-value below 0.05, which is the FDR analogue of the p-value.

We also calculated population differentiation using ARLEQUIN as described above but considering two subsets of SNPs a) outlier SNPs potentially under positive selection and b) neutral SNPs. Principal Coordinate Analyses (PCoA) were performed with GenAlEx vs 6.5^79^ using the genetic distances obtained from ARLEQUIN for all the loci and these two subsets of SNPs. Additionally, we computed for each SNP and population the frequency of the major allele, considering all samples, and represented them using a heatmap and a hierarchical dendrogram as implemented in the R function ‘heatmap.2’ of the package ‘gplots’^80^. This analysis was also done considering all SNPs and the two subsets above mentioned.

Finally, the 64 bp sequences containing all outlier SNPs potentially under selection were blasted against the genome of the Nile tilapia (*Oreochromis niloticus*), the only species with a reference genome that belongs to the same order (Perciformes) as our study species. We used the BLASTN search tool of the Ensembl website (www.ensembl.org). We set the sensitivity of the search tool to ‘Distant homologies’ in order to maximise the length of the matches considering that a certain level of divergence is expected given the phylogenetic distance between both species. We allowed a maximum E-value of 10^−3^ and considered only matches that included at least half of the 64 bp sequence of each SNP. Whenever a sequence yielded a match within a gene, the annotated function of this gene was searched at the UniProt database (www.uniprot.org). When a sequence yielded a match in an intergenic region, the closest gene was identified and also its function searched at the UniProt database.

## Additional Information

**How to cite this article**: Carreras, C. *et al*. Population genomics of an endemic Mediterranean fish: differentiation by fine scale dispersal and adaptation. *Sci. Rep.* **7**, 43417; doi: 10.1038/srep43417 (2017).

**Publisher's note:** Springer Nature remains neutral with regard to jurisdictional claims in published maps and institutional affiliations.

## Supplementary Material

Supplementary Tables and Figures

Supplementary Dataset 1

## Figures and Tables

**Table 1:** Sampling information.

	Karaburun Peninsula	Island of Vir	Tremiti Islands	Torre Guaceto	Otranto	Porto Cesareo	*Correlation*
Acronym	KAP	VIR	TRE	TOG	OTR	POC	
Country	Albania	Croatia	Italy	Italy	Italy	Italy	
Coordinates	40.188617N 19.494167E	44.328298N 15.029783E	42.138583N 15.523950E	40.716650N 17.800050E	40.109233N 18.519217E	40.195250N 17.917950E	
N	28	35	22	32	29	30	
Reads per individual	2,504,072	2,504,026	2,504,112	2,504,074	2,503,911	2,504,074	
Mapped reads per individual	426,859	427,151	394,954	378,587	287,733	377,310	
Polymorphic SNPs	3,552	3,714	3,084	3,699	3,319	3,626	0.896 (0.016)
H_o_	0.180	0.171	0.200	0.171	0.184	0.183	−0.934 (0.005)
H_e_	0.198	0.190	0.220	0.190	0.202	0.198	−0.950 (0.004)
A	1.854	1.892	1.767	1.889	1.852	1.871	0.957 (0.003)
H_o-c_	0.152	0.151	0.150	0.151	0.152	0.158	0.161 (0.760)
H_e-c_	0.168	0.169	0.166	0.169	0.167	0.172	0.595 (0.213)
A_r_	1.396	1.399	1.393	1.398	1.404	1.404	0.459 (0.360)

Sampled locations for *S. tinca* including the number of individuals (N), the mean number of reads per individual and population genetic diversity estimates. Mean observed heterozigosity (H_o_) and mean expected heterozygosity (H_e_) calculated considering within population polymorphic SNPs, mean number of alleles per locus (A), mean observed heterozigosity (H_o-c_) and mean expected heterozygosity (H_e.c_) corrected by the total number of SNPs and allelic richness (A_r_). The correlation column shows the results of the correlation tests of the diversity indexes to sample size (N) indicating the R value (p-value in brackets).

**Table 2:** Pairwise genetic distances among locations of *S. tinca* within the Adriatic using all 4,155 polymorphic SNPs.

	KAP	VIR	TRE	TOG	OTR	POC
KAP	—	0.0005	**0.0122**	**0.0022**	**0.0035**	**0.0032**
VIR	0.0012	—	**0.0111**	**0.0024**	**0.0038**	**0.0030**
TRE	**0.0220**	**0.0185**	—	**0.0069**	**0.0082**	**0.0073**
TOG	**0.0046**	**0.0041**	**0.0134**	—	**0.0010**	0.0006
OTR	**0.0058**	**0.0053**	**0.0138**	0.0016	—	**0.0010**
POC	**0.0054**	**0.0055**	**0.0141**	0.0012	0.0015	—

Below the diagonal F_ST-WC_ values and above the diagonal F_ST−RH_ values. Values in bold are those significant after FDR correction (for a P-value < 0.05, FDR = 0.0151). KAP: Karaburun Peninsula, Albania; VIR: Island of Vir, Croatia; TRE: Tremiti Islands, Italy; TOG: Torre Guaceto, Italy; OTR: Otranto, Italy and POC: Porto Cesareo, Italy.
