# Supplementary material for: Population genomics of an endemic Mediterranean fish: differentiation by fine scale dispersal and adaptation
Source: Sci Rep. 2017 Mar 6;7:43417. doi: 10.1038/srep43417 (PMC5338269; doi:10.1038/srep43417)
Supplement: Supplementary Tables and Figures [file srep43417-s1.pdf]

Population genomics of an endemic Mediterranean fish: differentiation by fine scale dispersal and adaptation

Carlos Carreras, Víctor Ordóñez, Lorenzo Zane, Claudia Kruschel, Ina Nasto, Enrique Macpherson, Marta Pascual

Supplementary Table S1: **Pairwise genetic distances among locations using outlier and neutral SNPs.**

F_{ST}	KAP	VIR	TRE	TOG	OTR	POC
**KAP**	-	0.0005	**0.0116**	**0.0023**	0.0026	**0.0032**
**VIR**	**0.0257**	-	**0.0094**	**0.0021**	**0.0028**	**0.0033**
**TRE**	**0.3389**	**0.2749**	-	**0.0064**	**0.0064**	**0.0066**
**TOG**	**0.1036**	**0.0655**	**0.2475**	-	0.0004	0.0003
**OTR**	**0.1704**	**0.1172**	**0.1885**	**0.0906**	-	0.0005
**POC**	**0.0952**	**0.2383**	**0.2383**	-0.0076	**0.0875**	-

Pairwise genetic distances ($F_{ST\text{-}WC}$) among locations of *S. tinca* within the Adriatic Sea, using the 19 outlier SNPs obtained through coalescent and Bayesian simulations (below the diagonal), and the 3,934 neutral SNPs (above the diagonal). Significant values after FDR correction in bold (for a P-value<0.05, FDR=0.0151). KAP: Karaburun Peninsula, Albania; VIR: Island of Vir, Croatia; TRE: Tremiti Islands, Italy; TOG: Torre Guaceto, Italy; OTR: Otranto, Italy and POC: Porto Cesareo, Italy.

***Supplementary Table S2*: Percentage of assignment of all locations of the 176 individuals of *Symphodus tinca* using all SNPs**

	Locations							Populations			
	**KAP**	**VIR**	**TRE**	**TOG**	**OTR**	**POC**	**Unassigned**	**East**	**South-west**	**Tremiti**	**Unassigned**
**KAP**	**28.6**	46.4	0.0	14.3	0.0	0.0	10.7	**78.6**	10.7	0.0	10.7
**VIR**	20.0	**51.4**	0.0	14.3	0.0	5.7	8.6	**68.6**	14.3	0.0	17.1
**TRE**	0.0	0.0	**95.5**	4.6	0.0	0.0	0.0	0.0	13.6	**68.2**	18.2
**TOG**	3.1	3.1	0.0	**46.9**	6.3	21.9	18.8	0.0	**93.8**	0.0	6.3
**OTR**	0.0	10.3	0.0	20.7	**41.4**	24.1	3.5	0.0	**100.0**	0.0	0.0
**POC**	0.0	3.3	0.0	33.3	0.0	**43.3**	20.0	0.0	**100.0**	0.0	0.0

Each cell contains the percentage of individuals sampled in a given location (row) that is assigned to each location or population (columns) according to the Bayesian analysis implemented in GeneClass2. Bold values represent self-assignments. The 'Unassigned' columns includes the percentage of individuals with an assignment score lower than 0.95. KAP: Karaburun Peninsula, Albania; VIR: Island of VIR, Croatia; TRE: Tremiti Islands, Italy; TOG: Torre Guaceto, Italy; OTR: Otranto, Italy and POC: Porto Cesareo, Italy. The three populations are defined based on the significance of pairwise comparisons (Fig. 4a) and include East Adriatic (KAP and VIR), South-west Adriatic (TOG, OTR and POC) and Tremiti Islands (TRE).

***Supplementary Table S3*: Results of the BLASTN analysis of the 12 outlier SNPs under positive selection**

SNP	Gene	Distance (bp)	Length (bp)	E-val	%ID	F_{ST-WC}	Function
**S1_6614124**	ENSONIT00000014653	71,199	64	6.00E-09	81	0.0559[A]	Uncharacterized protein
**S1_6225026**	flrt2	106,849	64	1.00E-10	86	0.3115[A,B]	cell adhesion
**S1_5791626**	tmem200a	7,868	57	8.00E-07	81	0.1062[A,B]	integral component of membrane
**S1_5429649**	pou3f3a	152,163	62	4.00E-14	92	0.1147[A,B]	transcription, DNA-templated
**S1_11289053**	CAAP1	32,052	63	3.00E-04	76	0.0844 [A]	apoptotic process
**S1_5467344**	ddx11	0	46	9.00E-04	83	0.0917[A]	sister chromatid cohesion
**S1_10077133**	zranb2	42,075	65	2.00E-09	85	0.0957[A]	regulation of transcription
**S1_2936757**	nrp1a	0	50	2.00E-06	84	0.0830[A]	vascular development
**S1_4450333**	nbeab	0	49	2.00E-08	90	0.0819[A]	Uncharacterized protein
**S1_2262059**	nptx2b	0	64	3.00E-07	78	0.0721[A]	regulation of circadian rhythm
**S1_9653836**	ENSONIT00000026164	8,580	64	8.00E-10	83	0.0731[A]	Uncharacterized protein
**S1_10260738**	PBX1	3,234	53	4.00E-05	83	0.0767[A]	transcription factor activity

Results with a match against the genome of the Nile tilapia (*Oreochromis niloticus*). For each SNP we provided: the closest gene in the Nile Tilapia genome, the minimum distance between the gene and the SNP (a distance of 0 indicates that the SNP is located within the gene), the length of the alignment, the E-value, the % Identity of the alignment, the overall F_{ST-WC} among populations for each specific locus and the function according to UniProt. The superscript letters on the F_{ST} values indicates whether an outlier SNP has been detected by A) ARLEQUIN or B) Bayescan.

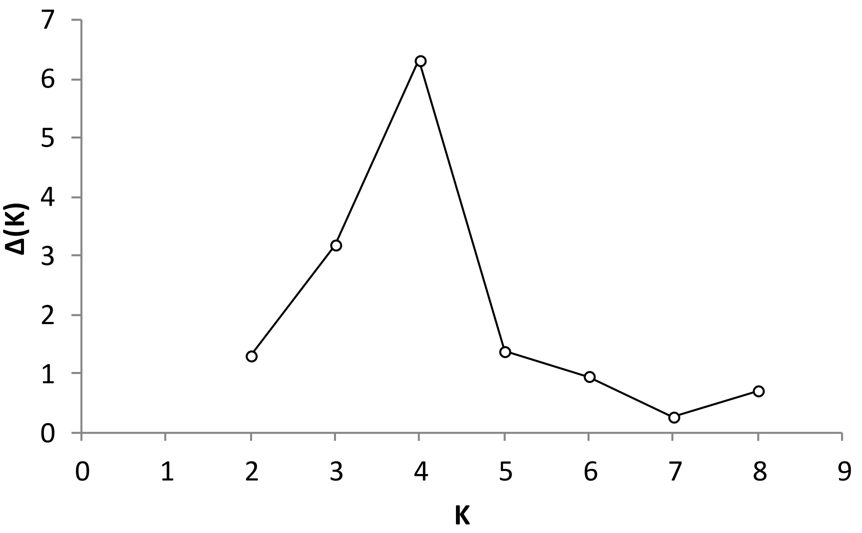

Supplementary Figure S1: Detection of the most likely number of genetic groups (K) using STRUCTURE and following the ad hoc statistic Δ(K) described in Evanno *et al.* 2005. The most likely number of genetic different groups was K=4.

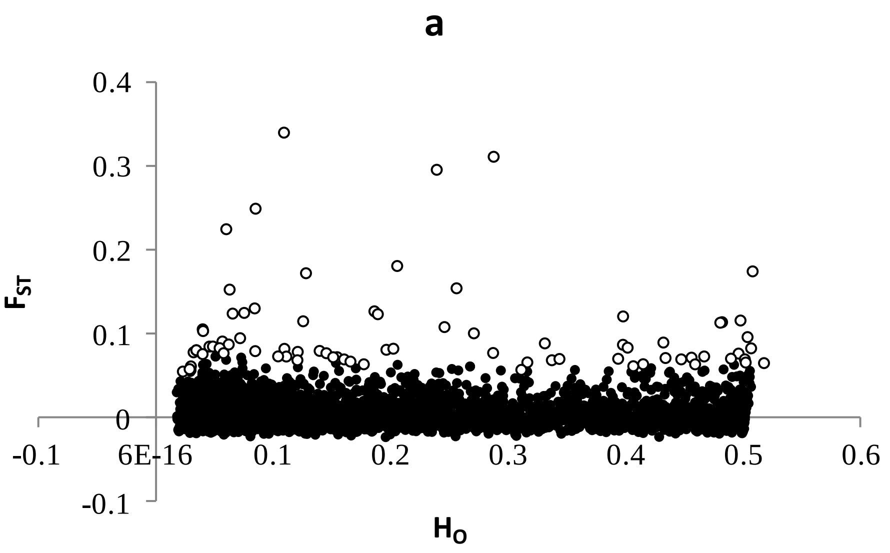

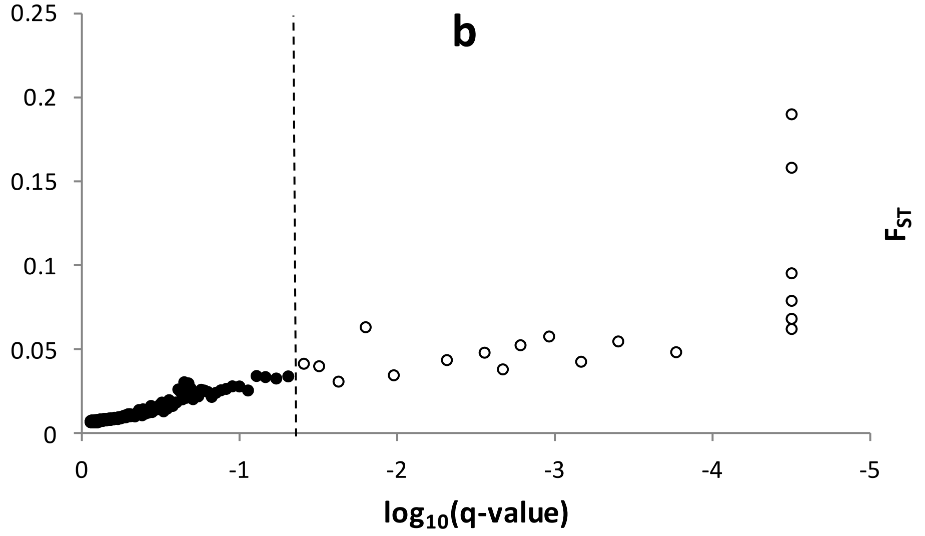

Supplementary Figure S2: Detection of outlier SNPs. a) Using coalescent simulations with ARLEQUIN: $F_{ST\text{-}WC}$ and observed Heterozygosities of all 4,155 SNPs are plotted and significant outlier SNPs shown as open circles (for a P-value<0.05, FDR=0.0054). b) Using bayesian methods with BAYESCAN: $F_{ST\text{-}WC}$ of all 4,155 SNPs are plotted against the log_{10} of the q-value. Significant outlier SNPs are shown as open circles. Dashed line represents the cut threshold of 5% of the q-value that delimits the outlier SNPs after FDR.

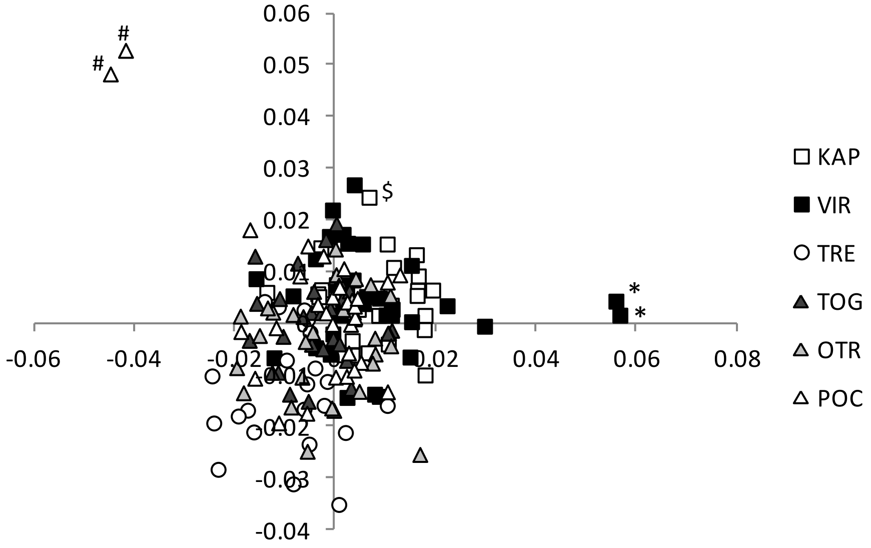

Supplementary Figure S3: MDS analysis of the 176 individuals of *Symphodus tinca* using neutral SNPs and including the divergent individuals from Vir (*), Porto Cesaeo (#) and Karaburun ($). KAP: Karaburun Peninsula, Albania; VIR: Island of VIR, Croatia; TRE: Tremiti Islands, Italy; TOG: Torre Guaceto, Italy; OTR: Otranto, Italy and POC: Porto Cesareo, Italy.

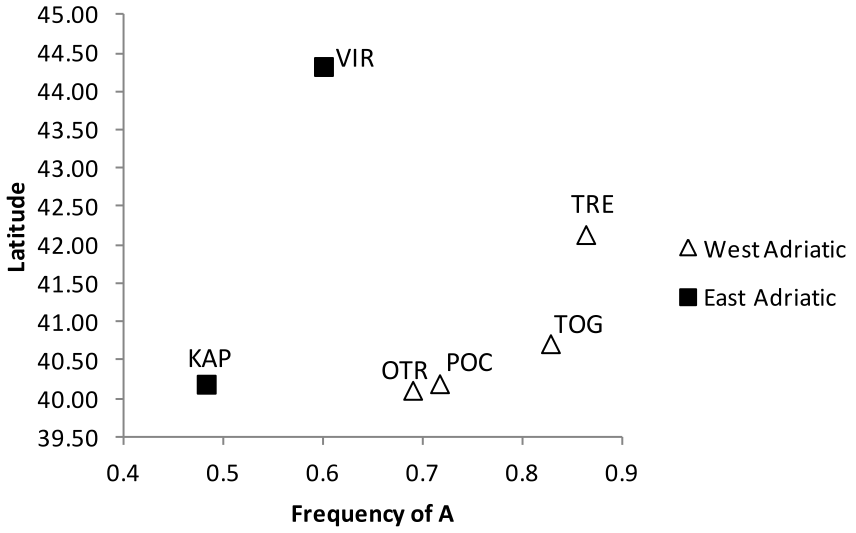

Supplementary Figure S4. Latitudinal variation of the frequency of the major allele (A) for the SNP S1_2262059, related to the circadian clock gene (nptx2b) allowing the lariat formation for the correct splicing of Intron 2. KAP: Karaburun Peninsula, Albania; VIR: Island of VIR, Croatia; TRE: Tremiti Islands, Italy; TOG: Torre Guaceto, Italy; OTR: Otranto, Italy and POC: Porto Cesareo, Italy.
